# Menopausal Status Modifies Breast Cancer Risk Associated with the Myeloperoxidase (*MPO*) *G463A* Polymorphism in Caucasian Women: A Meta-Analysis

**DOI:** 10.1371/journal.pone.0032389

**Published:** 2012-03-09

**Authors:** Noel Pabalan, Hamdi Jarjanazi, Lillian Sung, Hong Li, Hilmi Ozcelik

**Affiliations:** 1 School of Natural Sciences, Saint Louis University, Baguio City, Philippines; 2 Ontario Ministry of the Environment, Etobicoke, Toronto, Ontario, Canada; 3 Division of Hematology/Oncology, Hospital for Sick Children, Toronto, Ontario, Canada; 4 Fred A. Litwin Centre for Cancer Genetics, Samuel Lunenfeld Research Institute, Mount Sinai Hospital, Toronto, Ontario, Canada; Ohio State University Medical Center, United States of America

## Abstract

**Background:**

Breast cancer susceptibility may be modulated partly through polymorphisms in oxidative enzymes, one of which is myeloperoxidase (*MPO*). Association of the low transcription activity variant allele *A* in the *G463A* polymorphism has been investigated for its association with breast cancer risk, considering the modifying effects of menopausal status and antioxidant intake levels of cases and controls.

**Methodology/Principal Findings:**

To obtain a more precise estimate of association using the odds ratio (OR), we performed a meta-analysis of 2,975 cases and 3,427 controls from three published articles of Caucasian populations living in the United States. Heterogeneity among studies was tested and sensitivity analysis was applied. The lower transcriptional activity *AA* genotype of *MPO* in the pre-menopausal population showed significantly reduced risk (OR 0.56–0.57, p = 0.03) in contrast to their post-menopausal counterparts which showed non-significant increased risk (OR 1.14; p = 0.34–0.36). High intake of antioxidants (OR 0.67–0.86, p = 0.04–0.05) and carotenoids (OR 0.68–0.86, p = 0.03–0.05) conferred significant protection in the women. Stratified by menopausal status, this effect was observed in pre-menopausal women especially those whose antioxidant intake was high (OR 0.42–0.69, p = 0.04). In post-menopausal women, effect of low intake elicited susceptibility (OR 1.19–1.67, p = 0.07–0.17) to breast cancer.

**Conclusions/Significance:**

Based on a homogeneous Caucasian population, the *MPO G463A* polymorphism places post-menopausal women at risk for breast cancer, where this effect is modified by diet.

## Introduction

Myeloperoxidase (MPO) is a microbicidal enzyme secreted by reactive neutrophils at the sites of inflamed organs and tissues during the phagocytosis. Upon activation MPO catalyze the formation of powerful oxidants such as hypochlorous acid, which kills microbes. Levels of *MPO*-containing neutrophils are elevated in breast secretions as well as breast tissue with and without cancer [1], [2], [3]. It has been suggested that during chronic inflammation *MPO* is involved in DNA adduct formation through activation of heterocyclic amines to form chemically-reactive reactive oxygen species (ROS) in mammary epithelial cells [4]. Although ROS have important roles in cell signaling and homeostasis, the excess binds and damage DNA leading to oxidative stress, peroxidation of lipids and damage to cellular structures. In fact, inflammation and elevated peroxidase activity have been shown to increase the risk for women to develop breast cancer (relative risk 2.5, 95% confidence interval [CI] 1.01–5.16) [5]. An important neutralizer of the excess ROS is the consumption of antioxidants from fruits and vegetables. However, epidemiologic data regarding the association between fruit/vegetable intake and breast cancer risk were inconsistent [6]. The Long Island Breast Cancer Study Project showed that increased consumption of fruits and vegetables, rich sources of antioxidant nutrients which serve to reduce ROS levels, was associated with decreased breast cancer risk among post-menopausal but weaker associations among pre-menopausal women [7]. On the other hand, post-menopausal women with low levels of *MPO* activity who consumes low antioxidants sources are likely to have increased levels of oxidative stress [8] which may significantly raise breast cancer risk in this group [9].

A guanosine (*G*) to adenosine (*A*) nucleotide substitution, −*G463A* (rs2333227), located 463 bp upstream of transcription start site of *MPO* is found to have impact on the consensus transcription factor binding sites [10]. The commonly occurring *−463G* allele (frequency: ∼77%) were found to elevate *MPO* transcriptional activity, via promoting SP1 transcription factor binding whereas the minor *−463A* allele (frequency: ∼23%) was shown to confer ∼25 times lower transcriptional activation, leading to less inflammatory potential [10]. The high activity *−463G* allele has been associated with increased *MPO* activity in several diseases [11], [12] including lung cancer [13], [14]. The lower activity *A* allele which is associated with lower levels of polycyclic aromatic hydrocarbons [15] and ROS production elicited decreased risk in diseases such as coronary artery [16], Alzheimer's [12], multiple sclerosis [11], myeloid leukemia [17], esophageal [18] and lung cancers [14], [19], [20], [21]. Accumulating evidence also suggests association of *MPO-G463A* with breast cancer development although discrepancies exist.

In this study, we perform a meta-analysis to evaluate the association between the *MPO-G463A* variant and risk of breast cancer, also taking into consideration the potential modifying influences of menopausal status, antioxidant and vitamins/carotenoid intake of breast cancer and healthy women.

## Materials and Methods

### Selection of studies and genotype data

**Figure 1:**
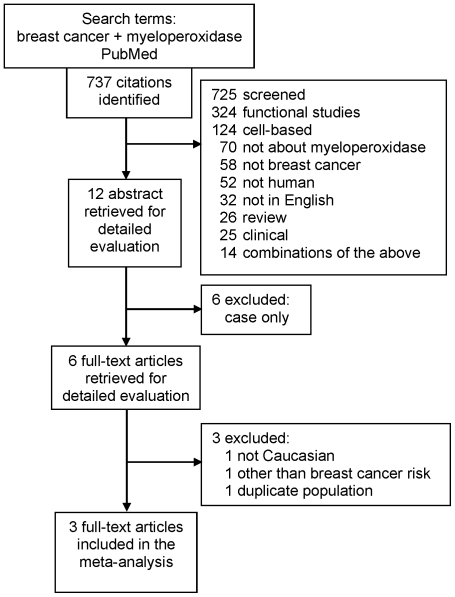
Summary of Literature Search.

Figure 1 shows the strategy used for PubMed search as of February 2011 yielding five articles that used Caucasians (living in the United States [US]) [9], [22], [23], [24], [25], after excluding one study that used Asian subjects [26]. Of the five, we also excluded another [23] given its focus on breast cancer recurrence and survival and not on risk. In two [9], [25] of the remaining four studies, overlapping data merited inclusion of only the most recent one [9]. One study [24] that investigated the *−764 T>C* (rs2243828) polymorphism was also included given its 100% genotype concordance in Caucasians (http://snp500cancer.nci.nih.gov) with *G463A* polymorphism. Thus, the final number of studies included in the meta-analysis was three [9], [22], [24] (Table 1). Two investigators independently verified for each article the demographic (first author's name, published year, country of origin, matching criteria) and the genotype data information. Sample sizes from these studies were derived from the genotypic data used to calculate summary effects for the *MPO G463A* polymorphism.

**Table 1:** Characteristics of the studies of *MPO-G463A* polymorphism and its association with breast cancer according to menopausal status.

	Pre-menopausal					Post-menopausal				
First Author (year)	Case	Control	Power = 0.05 OR>1.5	maf* in controls	HWE	Case	Control	Power = 0.05 OR>1.5	maf* in controls	HWE
Ahn (2004)	332	362	74.8	0.26	0.003	656	662	95.2	0.23	0.01
He (2009)	241	299	63.5	0.21	0.52	852	1,239	99.4	0.21	0.71
Li (2009)	---	---	---	---	---	894	865	98.7	0.28	0.06
Three studies	573	661	---	---	---	2,402	2,766	---	---	---

* maf: minor allele frequency; HWE: Hardy-Weinberg equilibrium.

### Primary analysis and subgroups

In the main analysis, we sought effects of the *MPO-G463A* polymorphism in pre-menopausal and post-menopausal women. There were three subgroups in our meta-analysis that involved the diet variable. One, genotypic data from the antioxidant subgroup were based on consumption of the combination of fruits and vegetables which were categorized as low and high. Two, genotypic data from the vitamin/carotenoid subgroup were based on low and high intake of vitamins C, E and carotenoids. Three, we investigated the level of antioxidant consumption patterns in menopausal women. In all analyses, the probability of differential risk associations between pre-menopausal and post-menopausal women as well as high and low consumption levels warranted testing for presence of interactions.

### Quality of studies and data analysis

Using the χ^2^ test, we evaluated deviation of the genotypic frequencies of control subjects from the Hardy-Weinberg equilibrium (HWE). While controls in Ahn *et al* [22] deviated from the HWE in the primary analysis and subgroups (Tables 1, 2 and 3), those in Li *et al* [9] did so only under the subgroup of post-menopausal women with low antioxidant intake (Table 3). Assuming an odds ratio (OR) of 1.5 at a genotypic risk level of α = 0.05 (two-sided), power was considered adequate at ≥80%. Statistical power of the studies was adequate for post-menopausal but not pre-menopausal women (Table 1). As well, data for the diet subgroup (Table 2) had adequate power to demonstrate an association, but not in the diet-menopausal subgroup (Table 3).

**Table 2:** Characteristics of the studies of *MPO-G463A* polymorphism and its association with breast cancer stratified by antioxidant and vitamin-carotenoid intake.

First Author (year)	Case	Control	Power = 0.05 OR>1.5	maf* in controls	HWE	Case	Control	Power = 0.05 OR>1.5	maf* in controls	HWE
	Antioxidant intake									
	Low					High				
Ahn (2004)	519	529	90.0	0.22	0.03	474	522	88.3	0.26	0.40
He (2009)	573	764	95.1	0.20	0.76	525	781	94.3	0.20	0.70
Two studies	1,092	1,293	---	---	---	999	1,303	---	---	---

	Carotenoid intake									
	Low					High				
Ahn (2004)	534	527	90.2	0.22	0.34	460	525	87.9	0.25	0.05
He (2009)	621	831	96.5	0.20	0.46	577	840	95.9	0.21	0.52
Two studies	1,155	1,358	---	---	---	1,037	1,365	---	---	---

**Table 3:** Characteristics of the studies of *MPO-G463A* polymorphism and its association with breast cancer stratified by menopausal status and antioxidant intake.

First Author (year)	Case	Control	Power = 0.05 OR>1.5	maf* in controls	HWE	Case	Control	Power = 0.05 OR>1.5	maf* in controls	HWE
	Antioxidant Intake									
	Low in pre-menopausal					High in pre-menopausal				
Ahn (2004)	150	180	43.8	0.24	0.05	174	176	46.2	0.27	0.03

	Low in post-menopausal					High in post-menopausal				
Ahn (2004)	372	338	75.7	0.2	0.52	274	315	67.6	0.26	0.87
Li (2009)	216	195	52.4	0.19	0.03	194	198	50.6	0.22	0.14
Two studies	588	533	---	---	---	468	513	---	---	---

All three studies [9], [22], [24] were matched by age. Two [9], [24] used date of blood collection and one [24] factored in menopausal status. In all, two [9], [24] of the three studies used a combination of the above-mentioned matching criteria. All P values were two-sided with significance set at <0.05 except in heterogeneity estimation. P values in the tests for interaction were corrected with the Bonferroni analysis. Data were analyzed using the G*Power statistical program (http://www.psycho.uni-dueldorf.de/aap/projects/gpower), Review Manager (RevMan 4.2; Cochrane Collaboration) and SigmaStat 2.03.

### Meta-analysis

We estimated OR and 95% CI of breast cancer associated with variant low activity compared with common high activity using the homozygous model (*AA* versus *GG*). We also examined the heterozygous genotype with low versus medium+high activity (*AA* versus *GA+GG*) as well as low+medium versus high activity (*AA+GA* versus *GG*). These contrasts correspond to recessive and dominant effects of the variant *A* allele, respectively. Finally, we estimated OR of the variant A allele frequency assuming the risk could differ across all three genotypes (co-dominant genetic model) [27]. To compare the OR on the same baseline, we used crude OR to conduct the meta-analysis. Pooled OR were obtained using either the fixed or random effects models. Fixed-effects was used in the absence of heterogeneity [28] while random-effects was used in its presence [29].

To test for robustness of the summary effects, we used sensitivity analysis which involved omitting one study at a time and recalculating the pooled OR. Heterogeneity between studies was estimated using the χ^2^-based Q test [30], significance set at P<0.10 [31]; explored using subgroup analysis [30] with menopausal status and diet as variables and quantified with the I^2^ statistic which measures degree of inconsistency among studies [32]. Publication bias was not investigated because of low sensitivity of qualitative and quantitative tests, the number of studies being lower than ten [33].

## Results

**Figure 2:**
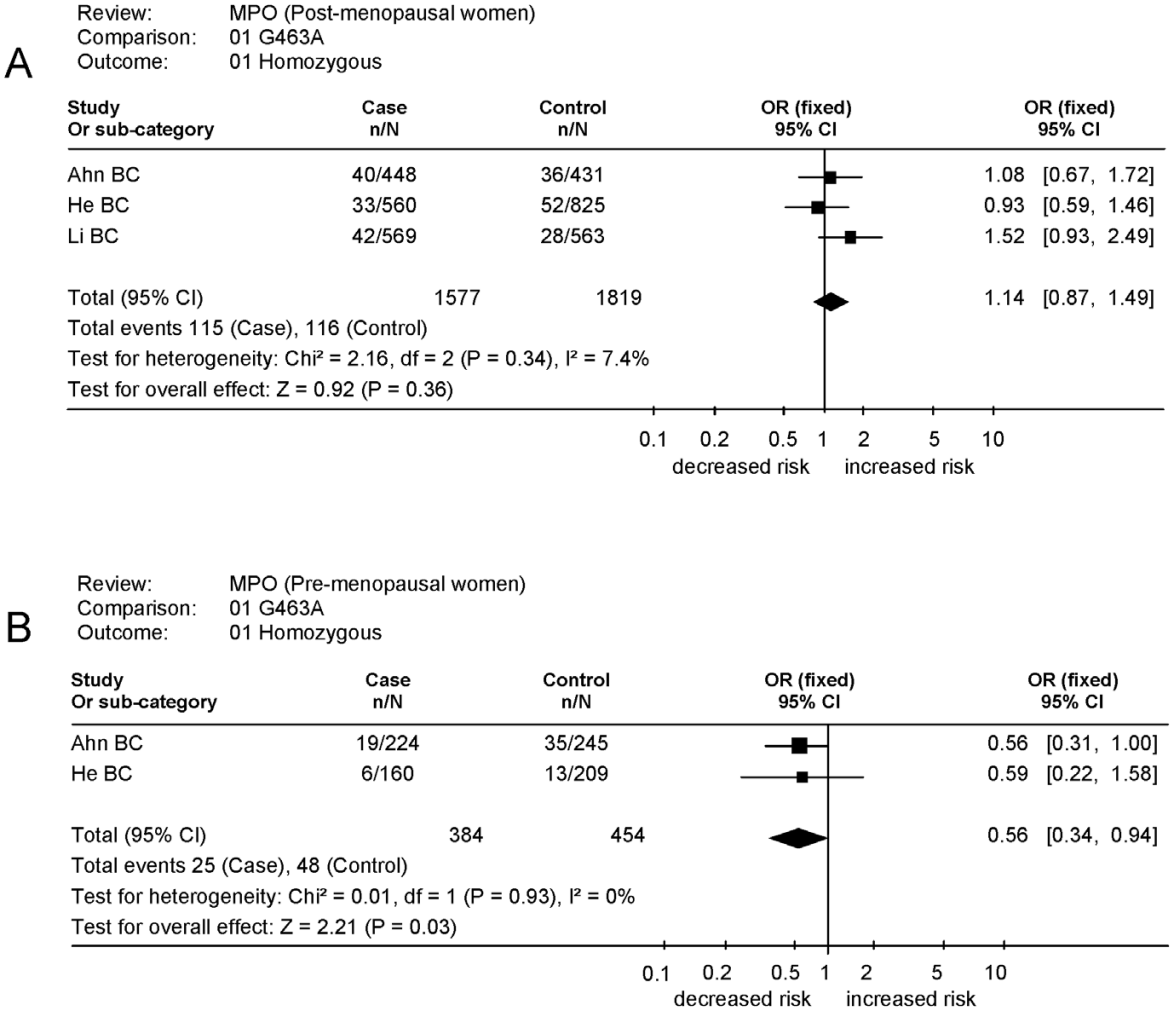
Forest plots of the odds ratios and confidence intervals of breast cancer associations in the homozygous model for (A) post-menopausal and (B) pre-menopausal women.

Here we investigated the breast cancer risk associated with *MPO-G463A* polymorphism status in ethnically homogenous Caucasian women. The post-menopausal (2,402 cases, 2,766 controls) and pre-menopausal (573 cases, 661 controls) groups came from three [9], [22], [24] and two studies [22], [24], respectively (Table 1). Initial meta-analysis has shown that post-menopausal women carrying the lower transcriptional *MPO* activity [*AA*] genotype were at non-significantly increased risk under homozygous and recessive models (OR 1.14, p = 0.35) (Table 4, Figure 2A). Under the same models, the pre-menopausal women carrying the lower transcriptional activity *AA* genotype, were found to be at significantly reduced risk (OR 0.56–0.57, p = 0.03) (Table 4, Figure 2B).

**Table 4:** Results of the meta-analysis for *MPO-G463A* polymorphism and breast cancer risk.

Updated 21 Nov 2011	Transcription Activity	OR (95% CI)	P value	P_het_	I^2^	OR (95% CI)	P value	P_het_	I^2^	P_interaction_ *
		N (cases/controls)				N (cases/controls)				
		Menopausal Status								
		Premenopausal 2 (573/661)				Postmenopausal 3 (2,402/2,766)				
A vs G	per allele effect	0.88 (0.72–1.06)	0.19	0.31	5	1.01 (0.95–1.12)	0.77	0.54	0	>1
AA vs GG	low versus high	**0.56 (0.34–0.94)**	**0.03**	0.93	0	1.14 (0.87–1.49)	0.36	0.34	7	0.32
AA vs GA+GG	low versus medium+high	**0.57 (0.34–0.93)**	**0.03**	0.99	0	1.14 (0.87–1.48)	0.34	0.38	0	0.36
AA+GA vs GG	low+medium versus high	0.94 (0.75–1.19)	0.62	0.34	0	1.02 (0.91–1.15)	0.68	0.31	14	>1

		Antioxidants Only								
		*Low 2 (1,092/1,293)*				*High 2 (999/1,303)*				
A vs G	per allele effect	*1.04 (0.91–1.20)*	*0.56*	*0.94*	0	**0.86 (0.74–0.99)**	**0.04**	0.10	62	>1
AA vs GG	low versus high	*1.05 (0.73–1.51)*	*0.80*	*0.95*	0	0.67 (0.45–1.01)	0.06	0.78	0	>1
AA vs GA+GG	low versus medium+high	*1.03 (0.72–1.48)*	*0.87*	*0.95*	0	0.70 (0.47–1.06)	0.09	0.97	0	>1
AA+GA vs GG	low+medium versus high	*1.05 (0.89–1.25)*	*0.54*	*0.96*	0	0.89 (0.75–1.05)	0.17	0.11	61	>1

		Carotenoids								
		*Low 2 (1,155/1,358)*				*High 2 (1,037/1,365)*				
A vs G	per allele effect	1.05 (0.92–1.20)	0.48	0.96	0	**0.86 (0.75–0.99)**	**0.03**	0.14	55	0.88
AA vs GG	low versus high	1.01 (0.70–1.47)	0.94	0.52	0	**0.68 (0.46–1.00)**	**0.05**	0.53	0	>1
AA vs GA+GG	low versus medium+high	0.99 (0.69–1.42)	0.96	0.47	0	0.71 (0.49–1.05)	0.09	0.66	0	>1
AA+GA vs GG	low+medium versus high	1.08 (0.91–1.26)	0.38	0.80	0	0.86 (0.73–1.01)	0.07	0.15	52	0.93

		Menopausal Status and Antioxidants								
		Premenopausal								
		*Low antioxidants 1 (450/180)*				*High antioxidants 1 (174/176)*				
A vs G	per allele effect	0.99 (0.69–1.42)	0.95	-----	-----	**0.69 (0.49–0.98)**	**0.04**	-----	-----	>1
AA vs GG	low versus high	0.74 (0.31–1.78)	0.51	-----	-----	**0.42 (0.18–0.97)**	**0.04**	-----	-----	>1
AA vs GA+GG	low versus medium+high	0.70 (0.30–1.65)	0.42	-----	-----	**0.42 (0.18–0.97)**	**0.04**	-----	-----	>1
AA+GA vs GG	low+medium versus high	1.08 (0.70–1.68)	0.73	-----	-----	**0.42 (0.18–0.97)**	**0.04**	-----	-----	0.98

		Postmenopausal								
		*Low antioxidants 2 (588/533)*				*High antioxidants 2 (468/513)*				
A vs G	per allele effect	1.21 (0.99–1.48)	0.07	0.77	0	1.06 (0.86–1.30)	0.60	0.83	0	>1
AA vs GG	low versus high	1.67 (0.96–2.88)	0.07	0.86	0	0.83 (0.46–1.51)	0.54	0.25	24	>1
AA vs GA+GG	low versus medium+high	1.61 (0.94–2.76)	0.08	0.91	0	0.89 (0.49–1.61)	0.70	0.34	0	>1
AA+GA vs GG	low+medium versus high	1.19 (0.93–1.51)	0.17	0.31	3	0.85 (0.66–1.09)	0.21	0.13	57	>1

OR (95% CI): odds ratio 95% confidence interval; P_het_: P value for heterogeneity; Given that all P values for the heterogeneity test were >0.10, the fixed-effects model was used;

* Bonferroni-corrected.

Removing the Ahn *et al* study [22], whose controls violated HWE did not change these risk effects by sensitivity analysis. All effects under menopausal status including outcomes of sensitivity analysis (data not shown) were obtained under homogeneous conditions (Table 4).

Table 4 shows subgroup antioxidant and carotenoid analyses indicating significantly reduced breast cancer risk in the co-dominant and homozygous models. This was observed in low activity *AA* genotype women (regardless of menopausal status) who consumed high levels of fruits-vegetables (OR 0.86, p = 0.04 and 0.67, p = 0.05). Separate analyses of fruits only and vegetables only yielded similar results (data not shown). Likewise, similar results were seen in such women with high levels of carotenoid intake (OR 0.86, p = 0.03 and 0.68, p = 0.05). Separate analyses of vitamins C and E yielded similar but non-significant results (data not shown).

Table 4 shows the protective role of high antioxidant intake, evident in the subgroup analysis by menopausal status. Thus, this level of antioxidant intake in women who carried the low activity *AA* genotype were protected from breast cancer risk, non-significant in post-menopausal (OR 0.83–0.89, p = 0.21–0.70) but significant in pre-menopausal (OR 0.42–0.69, p = 0.04) women. The pre-menopausal findings, however, came from just one study with a sample size of 350 [22]. Low levels of antioxidant consumption in post-menopausal women who carried the low activity *AA* genotype were associated with increased risk in all genetic models (OR 1.19–1.67, p values  = 0.07–0.17). Increased risk, however, was not evident in pre-menopausal women with low antioxidant intake.

Of the 32 comparisons in the primary and subgroup analyses in which tests for heterogeneity were applied, 22 (68.8%) had none (I^2^ = 0%). However, none of the tests of interaction between pre-menopausal and post-menopausal women as well as between low and high consumption in the subgroup analyses were significant after the Bonferroni correction treatment (Table 4).

## Discussion

### Menopausal Status

Our analysis has demonstrated that post-menopausal women carrying the low activity *AA* genotype were associated with non-significantly increased breast cancer risk (up to 1.1-fold) whereas the risk associated with pre-menopausal women who carried the low activity *AA* genotype was significantly protective (up to 44%). The altered breast cancer risk observed by menopausal status may be partly explained by the differences in age and levels of estrogen production between pre-menopausal and post-menopausal women [9]. Estrogen has been found to modify *MPO* activity levels by influencing gene expression, monocyte number, or degree of *MPO* release, potentially altering serum levels [34], [35], [36]. Estradiol levels was also shown to modulate the circulating *MPO* levels during the menstrual cycle [37]. More importantly, estrogen has been shown to differentially regulate *MPO* expression according to genotype [12].

A recent meta-analysis [38], which investigated risk associated with *MPO-G463A* polymorphism regardless of the menopausal status and ethnic background [22], [25], [26] reported no association with breast cancer. The strengths of our study include (a) ethnic (∼95% Caucasian) and geographical (USA) homogeneity; (b) the statistically significant pooled findings which were homogeneous (P_heterogeneity_ = 0.10–0.78) and (c) a substantial number of cases and controls were pooled from the studies, which significantly increased the statistical power of the analysis.

### Antioxidant Intake and Menopausal Status

An important modifier in the relationship between *MPO* genotype and breast cancer risk is consumption of fruits and vegetables. It has been shown that post-menopausal women with reduced levels of *MPO* activity who consume low antioxidants are likely to have increased levels of oxidative stress [8] which may significantly raise breast cancer risk [9]. Our findings also support this as the non-significantly increased risk effects of the post-menopausal women became significant (up to 1.7-fold) when they consumed low levels of antioxidants. On the other hand, post-menopausal women with low activity *MPO* genotype were found to be associated with statistically significant protective risk when they consumed high level of antioxidants. The analysis of antioxidant effects in pre-menopausal women have shown statistically significant protective effects (24–56%, up to p = 0.001) in all genetic models with high consumption of antioxidants, although these findings are based on one study. The relatively small sample size, particularly in the pre-menopausal group, may increase the likelihood of Type I error meriting caution regarding interpretation of its outcomes. The antioxidant intake data from two studies [9], [24] was collected prior to development of breast cancer, therefore misclassification bias between cases and controls is unlikely to affect the risk estimates.

### Gene-gene interactions

The modifying influences of diet, age and menopausal status are best considered in context of other genes in the oxidative stress pathway. Two studies in our analysis investigated the *MPO-G463A* polymorphism in concert with the variants of other antioxidant enzymes, including catechol-O-methyltransferase (*COMT*) [24], endothelial nitric oxide synthase (*NOS3*) heme-oxygenase-1 (*HO-1*) and catalase (*CAT*) [9]. Study-specific [24] joint effects of *COMT* and *MPO* was marginally protective (OR 0.28, 95% CI 0.08–1.00). In addition, the *CAT-MPO* combination may greatly decrease the hazard of death from breast cancer [39]. Available data on joint effects was not sufficient to allow further analysis of gene-gene interactions.

### Conclusion

Our meta-analysis implicates that menopausal status and intake of antioxidants modified the risk associated with breast cancer risk of women who carried the low activity *AA* genotype of *MPO-G463A* polymorphism. The non-significantly increased risk associated with post-menopausal women became highly significant when they consumed low levels of antioxidants. On the other hand, pre-menopausal women with the same lower activity genotype were at protective risk, which became more protective when they used high levels of antioxidants. Our findings suggest the role of estrogens which were shown to impact on the *MPO* activity. Future studies with larger sample sizes particularly among pre-menopausal women may shed light on complexities of the many pathways involved in oxidative stress and breast cancer development, providing hypotheses for future functional studies.
